# Coordinated Strategy for a Model-Based Decision Support Tool for Coronavirus Disease, Utah, USA

**DOI:** 10.3201/eid2705.203075

**Published:** 2021-05

**Authors:** Hannah R. Meredith, Emerson Arehart, Kyra H. Grantz, Alexander Beams, Theresa Sheets, Richard Nelson, Yue Zhang, Russell G. Vinik, Darryl Barfuss, Jacob C. Pettit, Keegan McCaffrey, Angela C. Dunn, Michael Good, Shannon Frattaroli, Matthew H. Samore, Justin Lessler, Elizabeth C. Lee, Lindsay T. Keegan

**Affiliations:** Johns Hopkins Bloomberg School of Public Health, Baltimore, Maryland, USA (H.R. Meredith, K.H. Grantz, S. Frattaroli, J. Lessler, E.C. Lee);; University of Utah, Salt Lake City, Utah, USA (E. Arehart, A. Beams, T. Sheets, R. Nelson, Y. Zhang, R.G. Vinik, D. Barfuss, J.C. Pettit, M. Good, M.H. Samore, L.T. Keegan);; Utah Department of Health, Salt Lake City (K. McCaffrey, A.C. Dunn);; Veterans Affairs Salt Lake City Health Care System, Salt Lake City (M.H. Samore, L.T. Keegan)

**Keywords:** COVID-19, coronavirus disease, SARS-CoV-2, severe acute respiratory syndrome coronavirus 2, viruses, respiratory infections, zoonoses, Utah, modeling

## Abstract

The coronavirus disease pandemic has highlighted the key role epidemiologic models play in supporting public health decision-making. In particular, these models provide estimates of outbreak potential when data are scarce and decision-making is critical and urgent. We document the integrated modeling response used in the US state of Utah early in the coronavirus disease pandemic, which brought together a diverse set of technical experts and public health and healthcare officials and led to an evidence-based response to the pandemic. We describe how we adapted a standard epidemiologic model; harmonized the outputs across modeling groups; and maintained a constant dialogue with policymakers at multiple levels of government to produce timely, evidence-based, and coordinated public health recommendations and interventions during the first wave of the pandemic. This framework continues to support the state’s response to ongoing outbreaks and can be applied in other settings to address unique public health challenges.

The emergence of severe acute respiratory syndrome coronavirus 2 (SARS-CoV-2) has demonstrated the need for epidemiologic models in public health decision-making. Modeling has been critical to planning outbreak responses since at least the emergence of HIV 40 years ago (*1*–*3*). However, the response to the coronavirus disease (COVID-19) pandemic has highlighted several challenges with incorporating modeling into public health decision-making.

The fast-moving operational timescales of public health policy are often at odds with the traditionally slower and iterative science of epidemiologic modeling. When models are effective, they catalyze policies that prevent their sometimes-dire predictions, thus making the initial predictions seem inaccurate. This feedback loop has heightened skepticism, resulting in high-profile controversies around modeling results (*4*,*5*).

In the rush to provide evidence-based guidance to policymakers, modeling experts were overwhelmed with requests, leaving little time to respond or to coordinate with broader efforts. Meanwhile, many groups unfamiliar with the nuances of how modeling has evolved through years of infectious disease modeling research were producing models for public policy that failed to reflect state-of-the-art modeling science (*6*,*7*). This situation often resulted in conflicting evidence presented to decision-makers tasked with quickly setting up pandemic response plans. As the pandemic has progressed, substantial efforts have been made to help stakeholders interpret the results and assumptions of multiple, often contradictory, modeling efforts for policy decisions. These efforts include proposed frameworks for effectively incorporating multiple models into a structured decision-making process (*8*) and efforts to assemble forecasts from multiple models to produce unified predictions as is done for many other common forecasting systems, such as weather forecasts (*9*).

A major challenge in developing evidence-based models for policy is aligning models with policymakers’ needs. Models that cannot rapidly provide actionable results, although useful in a basic science context, will not be useful for guiding policy. Likewise, not all models are equally well-equipped to answer every question, and aligning the best model to address a given policy question is challenging, especially during a rapidly evolving pandemic. This challenge is exacerbated by differing expectations between epidemiologists and policymakers. Epidemiologists often seek to match model assumptions to reality and highlight the resulting uncertainty, whereas policymakers seek a concrete basis for making and defending policy decisions and often need a single number to put the results into use (e.g., order a particular quantity of N95 masks). Developing strong relationships with policymakers is essential for clearly communicating this uncertainty.

As of June 22, 2020, the US state of Utah had a low attack rate (55 infections/10,000 population reported statewide, compared with 70 infections/10,000 population reported nationwide) and few deaths (158 deaths statewide, or 0.5 deaths/10,000 population, compared with 3.7 deaths/10,000 population nationwide), all accomplished with less aggressive mandated social distancing than other states. Utah’s success might be attributable to its early adoption of an integrated control strategy that has relied heavily on testing and isolating case-patients, contact tracing, and quarantining case-patient contacts (>300,000 persons tested statewide [936 tests/10,000 population] compared with 828 tests/10,000 population nationally). The decision to take this course, its implementation, and evaluation were informed heavily by an integrated modeling approach that brought together a diverse set of technical experts and public health and healthcare officials. Given the limited data on COVID-19 at the time, our approach was helpful for all involved; however, without a counterfactual scenario, we cannot determine whether our efforts had the intended consequences. With this caveat, we present the approaches taken over 3 different phases and highlight key points in hopes the lessons learned can inform future modeling efforts (Figure 1).

**Figure 1:**
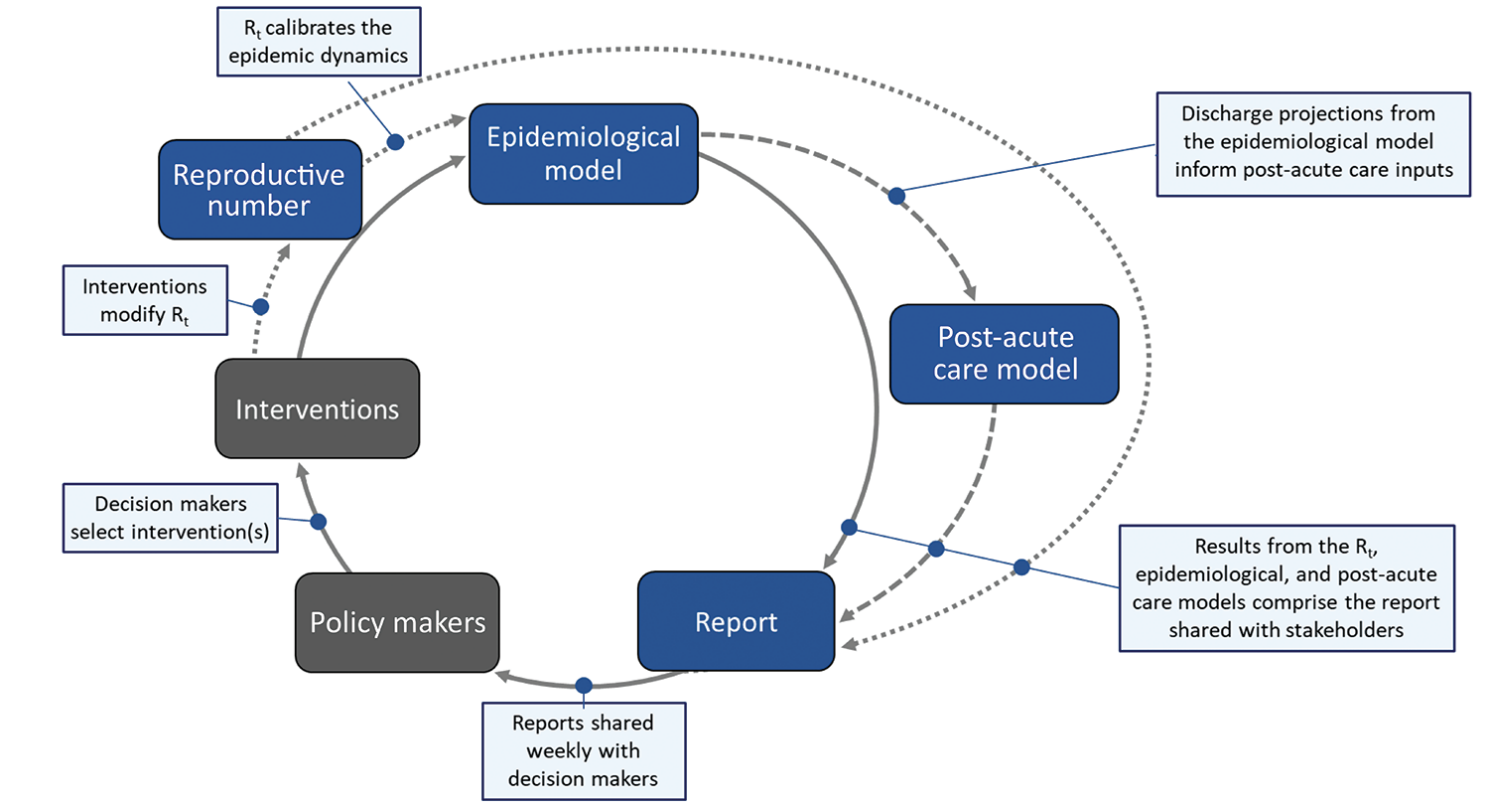
Schematic of the modeling process used as a decision support tool for coronavirus disease, Utah, USA. The epidemiologic model produces outputs of disease impact and key health outcomes that are used by the post–acute-care model. All model results are incorporated into the report, which is generated weekly and shared with policymakers who then make decisions on which interventions to implement. Those interventions impact the reproductive number, which is then used as an input to the epidemiologic model. The color of the box represents the time input was added, with dark blue for earliest and light blue for most recent. Policymakers and interventions are gray to indicate that although they are a critical component of our modeling process, they are external to our inputs to the process. R_t_, real-time effective reproduction number.

## Phase 1: Epidemiologic Model for Public Health Planning

**Figure 2:**
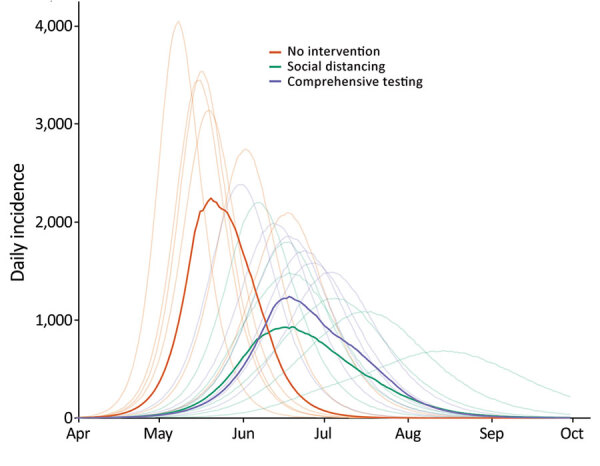
Example epidemiologic model output presented to stakeholders as part of decision support tool for coronavirus disease, Utah, USA. Model results compare daily incidence across 3 planning scenarios: no interventions, social distancing only, and comprehensive testing only. Bold lines represent the median daily incidence (cases/100,000 population) calculated from 1,000 simulations, whereas the lighter lines represent 15 random example simulations.

Utah, like other state, local, and national governments, sought epidemiologic modeling estimates to inform their COVID-19 response. Utah public health decision-makers initially engaged with our group, Infectious Disease Dynamics, at the University of Utah to help prepare for and respond to COVID-19. To address their questions, we adapted a metapopulation Susceptible-Exposed-Infectious-Recovered/Removed modeling process to develop planning scenarios for the state (J.C. Lemaitre et al., unpub. data, https://doi.org/10.1101/2020.06.11.20127894). We projected infections, deaths, and health system needs under multiple nonpharmaceutical interventions (NPIs) being considered by decision-makers (Figure 2). In particular, we compared the effects of comprehensive testing and isolation strategies on the lockdown measures being implemented by other states (e.g., California). Although testing and isolation strategies were not yet feasible in many states because of slow scale-up of testing capacity, Utah was well positioned to take such an approach. As of March 25, 2020, a national diagnostic medicine laboratory located in Salt Lake City had ample resources to rapidly develop and scale up COVID-19 testing capacity.

We compiled the model-based projections and comparison of NPIs and rapidly shared a report on March 23, 2020, with key leadership at the University of Utah Health, the Utah Department of Health (UDOH), ARUP Laboratories (Salt Lake City), the Governor’s Office of Management and Budget, and Intermountain Healthcare, the largest healthcare system in Utah. These stakeholders encompassed the key health decision-makers in the state, including those responsible for ≈60% of the state’s hospital market share.

On March 24, university leadership coordinated a meeting between scientists and policymakers to discuss this initial report. The goal of the meeting was to review model projections, compare the different NPI scenario estimates, and discuss the best paths forward for the state. The resulting consensus was that the state should strive to rapidly achieve levels of per-capita testing of symptomatic persons similar to those seen in South Korea, a goal that was achieved in Utah by March 25, 2020. After this meeting, we maintained open lines of communication with health experts and policymakers, soliciting insight into new operational questions (further discussed in phase 3) and distributing weekly scenario-based projections of probable outcomes under different NPIs over the course of the local outbreak.

## Phase 2: Establishing Local Model Consensus

**Figure 3:**
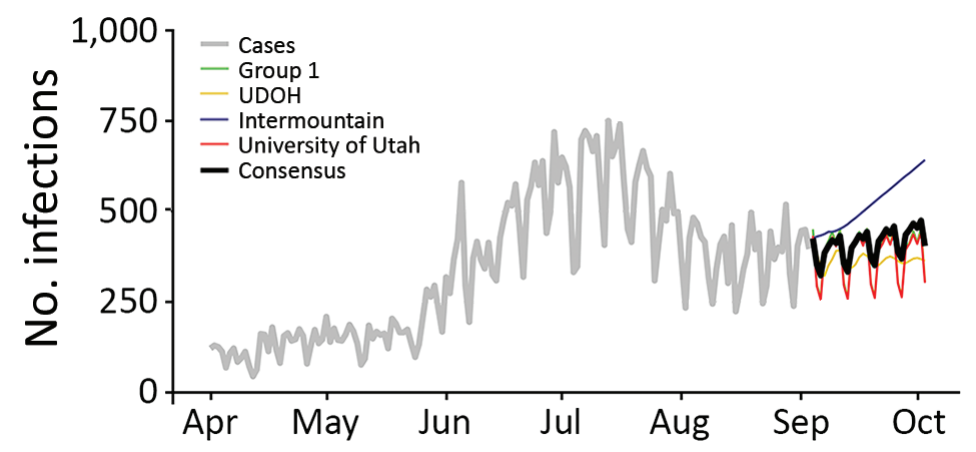
Example of a consensus model figure from a decision support tool for coronavirus disease, Utah, USA. Model results compare the number of new reported infections (daily) across the 4 modeling groups presented to Utah stakeholders on September 9, 2020. Light gray line represents reported infections, black line represents the consensus model (i.e., the average of the 4 individual group models), green line represents the results from modeling group 1, yellow line represents the results from the UDOH, blue line represents the results from the Intermountain Healthcare model, and red line represents the results from the University of Utah model. UDOH, Utah Department of Health.

The University of Utah model was not the only model used to estimate COVID-19 impact in Utah. In addition to national-level models that included projections for Utah (e.g., projections described in University of Washington Institute for Health Metrics and Evaluation [IHME] COVID-19 Health Service Utilization Forecasting Team et al., unpub. data, https://doi.org/10.1101/2020.04.21.20074732), 3 other groups within the state were developing models of COVID-19 to inform policy. Intermountain extended an existing Susceptible-Infected-Recovered (SIR) model to project expected burden on their healthcare facilities statewide, later switching to a timeseries model for short-term forecasting. UDOH used an SIR model, and another group constructed an operational model of COVID-19 that projected forward on the basis of current trends, thereby implicitly projecting the effect of current NPIs at the state level (group 1 in Figure 3), later moving to a timeseries model for short-term forecasting. The different modeling approaches, which often yielded qualitatively different results (Figure 3), were creating uncertainty about the relative strengths and weaknesses of policy options.

To improve consistency in model outputs and communication of results across the 3 modeling groups, we arranged weekly consensus modeling meetings starting on April 8, 2020, that included representatives from all groups and other stakeholders (e.g., UDOH). Those meetings covered evidence-based model parameters, key modeling scenarios (e.g., determining which NPIs to model), data quality, and appropriate interpretation of high-profile models from outside the state. At these meetings, participants learned that the University of Utah was using a Susceptible-Exposed-Infectious-Recovered/Removed model (later changing to a timeseries model for short-term forecasting) with a latent period of 5 days and an average duration of infection of 6 days, whereas Intermountain was using an SIR model with an average duration of infection of 6 days. Likewise, the University of Utah group assumed that 10% of all infections were in hospitalized case-patients and the duration of hospitalization was on average 11.5 days, whereas Intermountain assumed that 2.5% of infections were in hospitalized case-patients and the duration of hospitalization was on average 7 days. Further, the University of Utah assumed that 15% of hospitalized patients required a stay in the intensive-care unit (ICU), whereas Intermountain assumed that 38% of hospitalized patients required an ICU stay. The consensus modeling group also served as a forum for informal peer review of models from each group. The consensus modeling meetings produced weekly joint reports reflecting the collective research, modeling, and operational efforts of the group, standardizing the outputs (Figure 3) to improve communication. Central to these reports was presenting results from all 3 groups in a format that could enable comparisons, guide public health decision-makers on the strengths and limitations of each model type, and indicate which models were more appropriate for informing certain decisions, such as models that aimed to forecast weekly incidence compared with those aiming to provide big-picture epidemiologic dynamics. To improve communication, these reports began presenting a consensus model, which was calculated as the average of each of the individual group models over the forecast period.

## Phase 3: Iterative Modeling and Ongoing Assessment

As the epidemic evolved, new operational questions required new approaches. To address these new questions, we contacted collaborators at the University of Utah to develop new decision support tools that expanded the modeling process. In particular, assessing the efficacy of key interventions in a local context became paramount. Doing so required an increased focus on ensuring the model’s assumptions matched the current epidemic situation.

**Figure 4:**
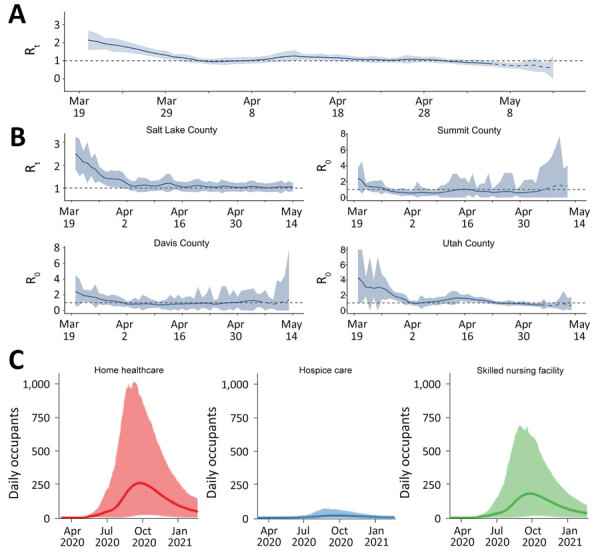
Sample model outputs from additional model components for a decision support tool for coronavirus disease, Utah, USA. Solid lines indicate the average daily occupancy, and shaded areas represent 95% CIs. A, B) Estimates of R_t_ for the entire state of Utah (A) and for 4 counties (B). The dashed blue line at the end of each time course represents the period within 1 serial interval from the end of the available data, where estimates of R_t_ are not accurate; dashed black line depicts R_t_ = 1, below which the disease will disappear and above which the disease will spread. C) Post–acute-care occupancy for each of 3 care types: home healthcare, hospice care, and skilled nursing facility. R_t_, real-time effective reproduction number.

To characterize the effectiveness of the NPIs that were implemented in Utah in March 2020, we estimated the time varying local reproduction number, R_t_ (the real-time average number of secondary infections from a single infected person), with assistance from the Study Design and Biostatistics Center at the University of Utah (Y. Zhang et al., unpub. data, https://doi.org/10.1101/2020.05.08.20095703). Estimates of R_t_ became a weekly input into the transmission model, and these projections served as a baseline for comparing current and possible interventions (Figure 4, panel A). As the epidemic progressed, local outbreaks sparked concerns of substantial spatial heterogeneity in the impact of interventions across the state. Hence, we began estimating R_t_ at the county level and capturing this heterogeneity in our wider modeling efforts, as well as including these estimates directly in the report beginning April 13, 2020.

As COVID-19 patients were discharged, public health officials learned that the pandemic would have downstream effects on post–acute-care facilities. These case-patients often require further supportive care after hospitalization; however, they might still be infectious and pose a risk to other long-term care facility residents and staff (*10*). One week after we shared model outputs with state decision-makers, the state opened a dedicated long-term care facility to accommodate COVID-19 patients. To help calibrate the appropriate capacity of the center and anticipate the need for expansion, we collaborated with a team of hospitalists (general internists who care for hospitalized patients) to extend the process with a module aimed at projecting post–acute-care flows. This module explicitly models the discharge of case-patients directly to home, to home healthcare, to skilled nursing facilities, or to hospice (Figure 4, panel B), and was first included in reports on May 18, 2020 (M. Maloney et al., unpub. data, https://doi.org/10.1101/2020.06.12.20129551).

## Phase 4: Ongoing Activities and Future Directions

Although we have devised a process for responding to the ongoing pandemic, the situation continues to evolve. What appears to be effective now might not continue to be fruitful as the outbreak progresses. Likewise, the questions that have arisen thus far represent just a small sample of the potential hurdles that might be faced during a dynamic situation. For instance, we are already working to integrate a health economic model with the post–acute-care components to help guide the development and expansion of additional patient-care resources after hospitalization. In addition, we are beginning to develop collaborations across states with similar experiences, priorities, and concerns to learn from their experiences and further improve pandemic response. Although some future directions are clear, others will emerge as the pandemic evolves. As subsequent outbreaks occur, the response continues to leverage these developed collaborations to provide the state with evidence-based guidance for pandemic response.

## Discussion

We identify 3 key points from the process so far. The first is establishing processes for bidirectional communication among stakeholders, the second is promoting communication and consensus among modeling teams, and the third is inviting multidisciplinary perspectives to inform modeling.

First, ongoing iterative communication with public health officials, policymakers, and other stakeholders is key for developing an understanding of policymakers’ needs and gaining their trust, thereby creating a bidirectional relationship with effective communication. Through the process of producing and sharing weekly scenario-based projections of outcomes with policymakers and health experts, we demonstrated that we incorporated their feedback into the model, offered new interventions and evaluation criteria to consider, and provided support in interpreting the projections. Regular, open communication between stakeholders and modelers also fostered an environment that facilitated conversation between modeling groups and spurred new modeling developments.

The second key point is that debate and discussion of results between modeling groups increased confidence in model results and overall interpretability by policymakers. Before Utah developed its own models, several high-profile, out-of-state models produced unrealistic projections because they failed to account for the local context. For instance, the IHME model predicted hospital capacity would be exceeded in early April, much earlier than was observed, probably a result of drawing parallels with other COVID-19 epidemics based on little evidence and failing to incorporate important contextual details (IHME COVID-19 Health Service Utilization Forecasting Team et al.). The guidance of local models produced a more measured approach to outbreak control (i.e., a rapid scale-up of state testing and isolation), compared with a strict lockdown, which would have been justified to prevent the dire hospital overflow predicted by other models**.** The interagency collaboration developed through the consensus group helped to draw on diverse perspectives, account for local context, and boost confidence in model projections statewide. Importantly, comparing multiple models helped refute the false narrative that differing models are necessarily in competition. This comparison helped to highlight to both the consumers of the results and the individual modeling teams that each model is a tool optimized for addressing a particular type of policy question by making certain assumptions.

Finally, modeling approaches need to be adaptable and multidisciplinary to address changing policy questions. By using a solution-oriented modular approach, we were able to adjust and expand the initial epidemiologic model to assess how using an NPI affected the number of cases, the number of hospital or ICU beds needed in the short term, and the number of skilled nursing facility beds needed on a longer time scale, as well as, ultimately, the effectiveness of the NPIs used. An additional benefit of incorporating multiple modeling components was the differing perspectives in evaluating model assumptions and interpreting outputs gained by collaborating with experts from a range of disciplines. This collaboration between epidemiologists, health economists, biostatisticians, and hospitalists yielded perspectives beyond any single discipline and enabled groups to focus on modeling within their areas of expertise. Each model component was developed as a separate module, but results were shared regularly to solicit feedback, determine how they would inform the other modules, and formulate a consistent message for stakeholders.

In conclusion, the framework we have described can be applied in other settings to address additional public health challenges. This approach is best used at the level that decisions are being made and policies put into place. Each jurisdiction, whether at the city, county, state, or regional level, has its own particular conditions that affect disease transmission and number of cases (e.g., population density and demographics), and which intervention and treatment options are feasible (e.g., local laboratory capacity to scale up testing). As a result, modeling approaches for the same public health threat are bound to vary. An interdisciplinary modeling hub with university-level support for these kinds of cross-cutting collaborations, such as the one we created, would enable the kind of inclusive, rigorous exchange that can yield valid models and estimates that multiple modeling groups can support. By enabling sharing of modeling approaches and sustaining dialogue focused on policymakers’ questions, the forum would help modelers propose relevant and operationalizable scenarios that will probably resonate with policymakers and result in greater uptake. Another strategy would be to apply this multidisciplinary approach at the national level; however, a continuous dialogue between modelers, experts on the varied local conditions, and local politicians would be integral for the success of a national-level response.
